# Partial activation of salt-inducible kinase 3 delays the onset of wakefulness and alleviates hypersomnia due to the lack of protein kinase A-phosphorylation site

**DOI:** 10.1093/sleep/zsae279

**Published:** 2024-12-04

**Authors:** Shinya Nakata, Tomoyuki Fujiyama, Fuyuki Asano, Haruna Komiya, Noriko Hotta-Hirashima, Motoki Juichi, Daiki Komine, Miyo Kakizaki, Aya Ikkyu, Seiya Mizuno, Satoru Takahashi, Chika Miyoshi, Hiromasa Funato, Masashi Yanagisawa

**Affiliations:** International Institute for Integrative Sleep Medicine (WPI-IIIS), University of Tsukuba, Tsukuba, Ibaraki, Japan; International Institute for Integrative Sleep Medicine (WPI-IIIS), University of Tsukuba, Tsukuba, Ibaraki, Japan; International Institute for Integrative Sleep Medicine (WPI-IIIS), University of Tsukuba, Tsukuba, Ibaraki, Japan; International Institute for Integrative Sleep Medicine (WPI-IIIS), University of Tsukuba, Tsukuba, Ibaraki, Japan; International Institute for Integrative Sleep Medicine (WPI-IIIS), University of Tsukuba, Tsukuba, Ibaraki, Japan; International Institute for Integrative Sleep Medicine (WPI-IIIS), University of Tsukuba, Tsukuba, Ibaraki, Japan; International Institute for Integrative Sleep Medicine (WPI-IIIS), University of Tsukuba, Tsukuba, Ibaraki, Japan; International Institute for Integrative Sleep Medicine (WPI-IIIS), University of Tsukuba, Tsukuba, Ibaraki, Japan; International Institute for Integrative Sleep Medicine (WPI-IIIS), University of Tsukuba, Tsukuba, Ibaraki, Japan; Laboratory Animal Resource Center and Transborder Medical Research Center, University of Tsukuba, Tsukuba, Ibaraki, Japan; Laboratory Animal Resource Center and Transborder Medical Research Center, University of Tsukuba, Tsukuba, Ibaraki, Japan; International Institute for Integrative Sleep Medicine (WPI-IIIS), University of Tsukuba, Tsukuba, Ibaraki, Japan; International Institute for Integrative Sleep Medicine (WPI-IIIS), University of Tsukuba, Tsukuba, Ibaraki, Japan; Department of Anatomy, Graduate School of Medicine, Toho University, Tokyo, Japan; International Institute for Integrative Sleep Medicine (WPI-IIIS), University of Tsukuba, Tsukuba, Ibaraki, Japan; Department of Molecular Genetics, University of Texas Southwestern Medical Center, Dallas, TX, USA; Life Science Center for Survival Dynamics, Tsukuba Advanced Research Alliance, University of Tsukuba, Tsukuba, Ibaraki, Japan

**Keywords:** NREM sleep, REM sleep, Circadian behavior, SIK3, kinase

## Abstract

**Study Objectives:**

Sleep/wakefulness is regulated by intracellular signaling pathways composed of protein kinases such as *salt-inducible kinase 3* (*Sik3*). *Sik3*-deficiency in neurons decreases nonrapid eye movement (NREM) sleep time and electroencephalogram (EEG) delta power during NREM sleep, while *Sik3*^*Slp*^ mice lacking a protein kinase A (PKA)-phosphorylation site, S551, show hypersomnia phenotype. In this study, we examined how a phosphomimetic mutation of the 221st threonine residue (T221E), which provides a partial (weak) constitutive activity of the kinase, affects sleep/wakefulness and circadian behavior. We also examined the effect of *T221E* substitution on the hypersomnia phenotype of *Sik3*^*Slp*^ mice.

**Methods:**

We examined the sleep/wake behavior of heterozygous and homozygous *Sik3*^*T221E*^ mice and *Sik3*^*T221E;Slp*^ mice using EEG and electromyogram recording. We also examined the circadian behavior of *Sik3*^*T221E*^ mice using a running wheel under the light–dark cycle and constant darkness.

**Results:**

Heterozygous and homozygous *Sik3*^*T221E*^ mice showed normal sleep time and sleep homeostatic responses. Homozygous *Sik3*^*T221E*^ mice exhibited a delayed onset of wakefulness at the early dark phase and longer circadian periods. *Sik3*^*T221E;Slp*^ mice showed decreased NREM sleep time and homeostatic responses compared to *Sik3*^*Slp*^ mice.

**Conclusions:**

Our results suggest that the peak onset of wakefulness is sensitive to disturbed kinase activity of SIK3, and the relationship between phosphorylation at T221 and S551 is critical for regulating sleep need.

## Graphical Abstract

**Graphical Abstract:**
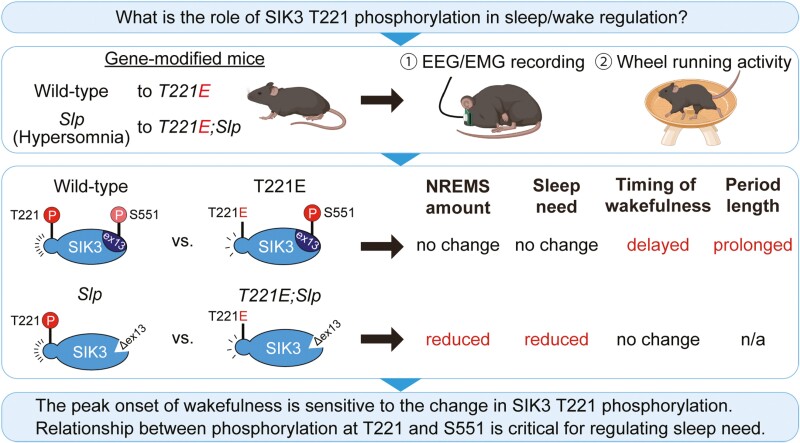

**Statement of Significance**

SIK3 is a key molecule regulating sleep propensity under sleep homeostatic and circadian control. The phosphorylation of T221 in SIK3 is required for its kinase activity and for enhancing sleep quantity and quality. However, the role of the phosphorylation of T221 in the sleep homeostatic and circadian regulation for proper sleep/wakefulness remains largely unknown. Here, we demonstrated that the introduction of *T221E* substitution did not significantly affect sleep/wakefulness but affected circadian-related behavior. The proper kinase activity is required for hypersomnia phenotype in *Sik3*^*Slp*^ mice. This study suggests that modulating SIK3 kinase activity can be a therapeutic target for hypersomnia and morning wake disturbances.

## Introduction

**Figure 1.**
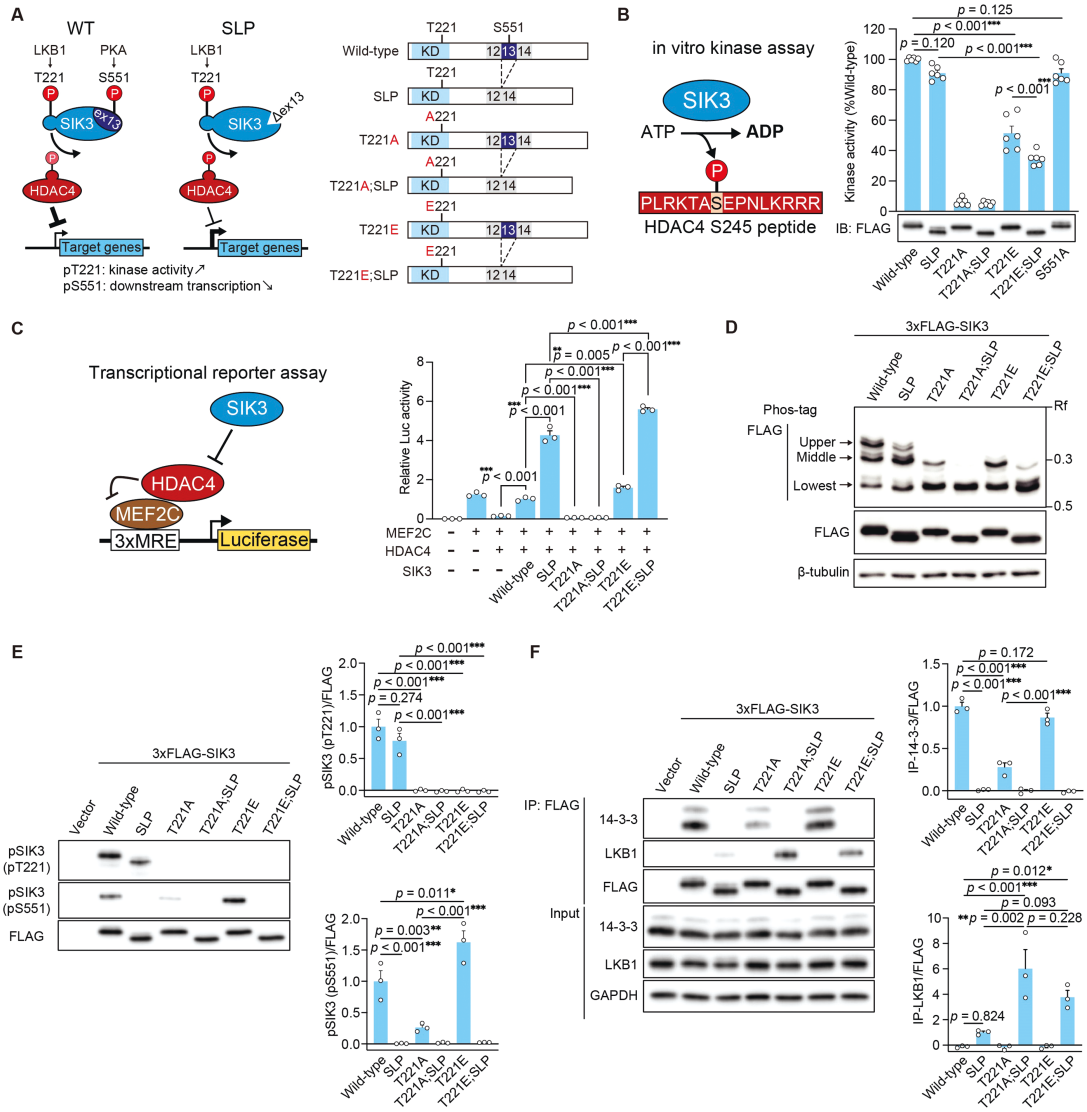
SIK3 (T221E) has distinct characteristics in kinase activity and transcription. (A) Schematic diagram of the SIK3 signaling pathway (left) and the structure of SIK3 variants (right). LKB1 phosphorylates T221 in the kinase domain (KD) of SIK3 to activate the kinase activity while PKA phosphorylates S551 in the exon13-encoded region of SIK3 to suppress the downstream transcription. Loss of the S551 phosphorylation enhances the downstream transcription and promotes sleep. (B) In vitro kinase assay for SIK3 variants expressed in HEK293T cells. The kinase activity was calculated based on ADP consumed for substrate phosphorylation. The western blotting result indicates that a similar amount of the SIK3 variants was used in the subsequent kinase assay. (C) Luciferase transcriptional reporter assay using HEK293T cells expressing the indicated proteins. The transcriptional activity was determined based on the luminescence emitted by the transcribed luciferases. (D) Immunoblots following Phos-tag SDS-PAGE of 3xFLAG-SIK3 variants-expressing HEK293T cells. Anti-FLAG antibody was used for the detection of SIK3 following both Phos-tag SDS-PAGE and SDS-PAGE, and β-tubulin was used as a loading control. Arrows indicate three main phosphorylation statuses: upper, middle, and the lowest. Rf values indicate the degree of migration. (E) Immunoblots of 3xFLAG-SIK3 variants-expressing HEK293T cells for the phosphorylation status in SIK3 (left). Quantification of phosphorylated SIK3 (phosphorylated T221: pT221) or phosphorylated SIK3 (phosphorylated S551: pS551) over FLAG band intensity (right). (F) Immunoblots of SIK3 variants immunoprecipitated (IP) from transfected HEK293T cells using anti-FLAG antibody (left). The immunoprecipitates were subjected to immunoblotting using anti-14-3-3, anti-LKB1, and anti-FLAG. Quantification of co-immunoprecipitated 14-3-3 or LKB1 over FLAG band intensity (right). One-way ANOVA followed by Tukey’s test (B, C, E, F). Data are presented as the mean ± SEM. *p < 0.05, **p < 0.01, ***p < 0.001.

Sleep is a fundamental behavior that is observed in a wide variety of animals from humans to hydra [1, 2], and is necessary to keep the brain and body in proper and healthy condition [3, 4]. Recent studies identified protein kinases and transcription factors involved in the regulation of sleep/wakefulness [5–12]. Salt-inducible kinase 3 (SIK3) was identified as a sleep-regulating molecule through a forward genetic study [5]. *Sik3*^*Slp*^ mice, which express SIK3 (SLP) proteins lacking exon13-encoded region due to a splice mutation, exhibit long nonrapid eye movement sleep (NREMS) time and high electroencephalogram (EEG) delta density during NREMS [5, 13], a commonly used indicator of sleep need or quality [14, 15]. *Sik3*^*Slp*^ mice work as a murine model of idiopathic hypersomnia based on short sleep latency and response to pharmacological intervention on hypersomnia [16]. The exon13-encoded region lacking in SIK3 (SLP) proteins contains a conserved protein kinase A (PKA) site, the 551st serine residue (S551) [5]. Phosphodeficient S551A mutation and partially phosphomimetic S551D mutation were both found to enhance NREMS time and delta density [13]. Additionally, these mutations resulted in a loss of binding affinity to 14-3-3 protein [13, 17, 18] and promoted the downstream transcription [8, 18]. These findings indicate that PKA negatively regulates SIK3 activity in relation to the downstream transcription through phosphorylation at S551 and 14-3-3 binding, which contributes to the suppression of NREMS time and delta density [8] (Figure 1A). Besides NREMS regulation, SIK3 is involved in the regulation of REMS time and EEG theta power during REMS [5, 8, 19]. Furthermore, the presence of SIK3 in the suprachiasmatic nucleus (SCN) is necessary for a rapid and robust increase in wakefulness at the beginning of the dark phase, and the loss of SIK3 in the SCN resulted in a prolonged period length under constant darkness [20].

In addition to PKA, liver kinase B1 (LKB1) phosphorylates SIK3 at 221st threonine residue (T221) within the kinase domain and its phosphorylation is required for the SIK3 kinase activity [21, 22] (Figure 1A). The findings that loss of LKB1 in neurons resulted in a decrease in NREMS time and delta density [7, 8], along with the observation that phosphorylation at the T221 was enhanced following sleep deprivation [5], suggest that LKB1-mediated T221 phosphorylation plays a role in promoting sleep. Consistently, mice with a heterozygous kinase-dead T221A mutation exhibited a decreased NREMS delta density with normal NREMS time [8] although wild-type SIK3 allele may compensate for the mutant T221A allele. Thus, homozygous T221A mutant mice are crucial to fully elucidate the role of T221 phosphorylation in sleep regulation. However, mice with homozygous T221A exhibited growth retardation similar to *Sik3*-deficient mice [8, 23], rendering them unsuitable for assessing sleep/wakefulness. In contrast to the phosphodeficient T221A mutation, SIK3 protein harboring the phosphomimetic T221E substitution exhibits moderate constitutive kinase activity that is independent of LKB1 regulation [8, 21, 24], which may facilitate further investigation into the role of T221 phosphorylation in sleep/wake regulation.

Regarding the relationship between the phosphorylation at S551 and T221 in SIK3, the enhancement of the downstream transcription and the hypersomnia phenotype in *Sik3*^*Slp*^ mice were abolished by the introduction of the T221A mutation into the *Slp* allele [8], suggesting that the gain-of-function effect of the *Slp* or S551A mutation depends on the T221 phosphorylation and the SIK3 kinase activity [8, 19]. In other words, SIK3 kinase activity serves as a convergent output for the upstream signaling pathways mediated by LKB1 and PKA (Figure 1A), and T221A substitution negates the effect of both LKB1 and PKA. T221E substitution, either the presence or absence of the *Slp* mutation, may allow us to investigate the additional relationship of the two phosphorylation sites, T221 and S551, in the modulation of sleep/wakefulness.

Here, we examined sleep/wakefulness and circadian behavior of heterozygous and homozygous *Sik3*^*T221E*^ mice. We also examined whether the presence of *T221E* on the *Slp* allele affects a long-sleeper phenotype of *Sik3*^*Slp*^ mice to clarify the relationship of two phosphorylation sites on sleep. Homozygous *Sik3*^*T221E*^ mice showed sleep/wakefulness similar to wild-type mice but with a delayed increase in wakefulness at the beginning of the dark phase and prolonged period length under constant darkness. NREMS time of *Sik3*^*T221E;Slp*^ mice was longer than *Sik3*^*T221E*^ mice and shorter than *Sik3*^*Slp*^ mice. Thus, our study revealed that the onset of the peak of wakefulness is influenced by disturbed kinase activity of SIK3, and the relationship between phosphorylation at T221 and S551 controls sleep need.

## Methods

### Mice

Mice were housed under controlled temperature and humidity conditions and were maintained on a 12-hour light:12-hour dark cycle. Food and water were provided to mice ad libitum. The background of all mice used for phenotypic analyses in this study was C57BL/6N (CLEA Japan). All animal experimental procedures were approved (Approved protocol ID #20-276) and conducted following the guidelines established by the Institutional Animal Care and Use Committee of the University of Tsukuba. Male mice were used in this study because female mice show a smaller response in EEG delta power and NREMS time after sleep deprivation [25].

### Generation of *Sik3*^*T221E*^ and *Sik3*^*T221E;Slp*^ mice

To generate *Sik3*^*T221E*^ and *Sik3*^*T221E;Slp*^ mice, we used the methodology previously described [26] with some modifications. For utilization of CRISPR/Cas9 system, we designed guide RNA (gRNA) and donor oligonucleotide. The sequence of gRNA synthesized using the GeneArt Precision gRNA Synthesis Kit (ThermoFisher) was 5ʹ-CAGGGCAGCTGCTGAAGACG-3ʹ. The donor oligonucleotide was 200 bases including the sequence to introduce threonine to glutamic acid substitution and several silent mutations to disrupt the targeting sequences. The gRNA, donor oligonucleotide, and GeneArt Platinum Cas9 Nuclease (ThermoFisher) were electroporated into *Sik3*^*Slp/+*^ zygotes using a NEPA 21 electroporator (NEPAGNENE). The next day, the electroporated embryos, which developed to the two-cell stage, were transferred into pseudopregnant ICR mice (Jackson Laboratory Japan). The genomic DNA sequence of F0 mice for *T221E* allele in *Sik3* was examined by genotyping PCR and direct sequencing. To extract *Sik3*^*T221E*^ mice line and *Sik3*^*T221E;Slp*^ mice line, respectively, F0 mice were crossed with C57BL/6N mice, and then the genomic DNA sequence of F1 mice for both *T221E* allele and *Slp* allele in *Sik3* was examined by genotyping PCR and direct sequencing for the confirmation. We performed the dCAPS method for the genotyping [27]. For the genotyping of the *T221E* allele in *Sik3*, the genomic DNA was amplified using T221E-Fw (5ʹ-CTCAGATGGTTCACTGCCTACTTCCT-3ʹ) and T221E-Rv (5ʹ-CGGCATAGGGAGGGCTGCCACAGTAC-3ʹ) to produce a 200-bp fragment and then digested with ScaI to produce 24-bp and 176-bp fragments when the allele was *T221E* allele. For the genotyping of the *Slp* allele in *Sik3*, the genomic DNA was amplified using Slp-Fw (5ʹ-AAAACCGTCCCACTTGTCACCATGACACCACTGA-3ʹ) and Slp-Rv (5ʹ-CTAGAGAGCACTGCCCGTGT-3ʹ) to produce a 228-bp fragment and then digested with DdeI to produce 33-bp and 195-bp fragments when the allele was wild-type allele.

### Cell culture and plasmids for transfection

HEK293T cells were obtained from the RIKEN BRC Cell Bank. The HEK293T cells were cultured in Dulbecco’s modified Eagle medium (Wako) supplemented with 10% fetal bovine serum (CORNING) and 1% penicillin/streptomycin (Wako) at 37°C in 5% CO_2_.

For kinase assay, Phos-tag SDS-PAGE, and immunoprecipitation assay, 3xFLAG-tagged SIK3 variants were inserted into the pcDNA3.1 vector (Thermo Fisher Scientific Inc.). When cells were grown to 70%–80% confluency, HEK293T cells were transfected with the pcDNA3.1 for 3xFLAG-SIK3 (wild-type), 3xFLAG-SIK3 (SLP), 3xFLAG-SIK3 (T221A), 3xFLAG-SIK3 (T221A;SLP), 3xFLAG-SIK3 (T221E), 3xFLAG-SIK3 (T221E;SLP), and/or 3xFLAG-SIK3 (S551A) using PEI Max (Polysciences) for kinase assay or Lipofectamine LTX with Plus Reagent (Thermo Fisher Scientific Inc.) for Phos-tag SDS-PAGE and immunoprecipitation assay according to the manufacturer’s protocols.

For luciferase reporter assay, mCherry-tagged SIK3 variants and HA-tagged MEF2C-VP16 were inserted into pcDNA3.1 vector and HDAC4 (wild-type) was inserted into pcAcGFP-C1 vector (Takara). When cells were grown to 70%–80% confluency, HEK293T cells were transfected with the 3xMRE-luciferase expression vector, phRL-SV40, pcDNA3.1 for HA-MEF2-VP16 and mCherry-SIK3 (wild-type), mCherry-SIK3 (SLP), mCherry-SIK3 (T221A), mCherry-SIK3 (T221A;SLP), mCherry-SIK3 (T221E), and mCherry-SIK3 (T221E;SLP) using FuGENE HD transfection reagent (Promega) according to the manufacturer’s protocol.

### In vitro kinase assay

Twenty-four hours after transfection, cells were processed with lysis buffer (20 mM HEPES, pH 7.5, 100 mM NaCl, 10 mM Na_4_P_2_O_7_, 1.5% Triton X-100, 15 mM NaF, 1× PhosSTOP [Roche], 5 mM EDTA, 1× protease inhibitor [Roche]). The lysates were rotated for at least 15 minutes at 4°C, and then centrifuged at 14 000 *g* at 4°C for 12 minutes. The supernatant was either used in kinase reaction or processed with 6× sample buffer for western blotting. We confirmed the expression level of the SIK3 mutant protein by western blotting to equalize the protein amount among samples. Then, equal amounts of SIK3 mutant proteins from the supernatant were subjected to immunoprecipitation using anti-Flag M2 magnetic beads (Sigma-Aldrich) at 4°C for 2 hours. In vitro kinase assay for SIK3 was performed using an ADP-Glo kinase assay kit (Promega) according to the manufacturer’s procedure with some modifications. After washing the beads with phosphate-buffered saline, the magnetic beads SIK3 bound to were incubated with ATP (100 μM), 1× kinase buffer, and HDAC5tide [28] (200 μM), equivalent to mouse HDAC4 (S245), as a substrate at room temperature for 1 hour. Then, ADP-Glo reagent was added to the mixture and incubated at room temperature for 40 minutes. Finally, kinase detection reagent was added to the mixture and incubated at room temperature for 40 minutes without light exposure, and the luminescence was measured using ARVO X5 (PerkinElmer). After the subtraction of the luminescence of the negative control (vector only) sample from the luminescence of the SIK3 variant samples, the kinase activity of each sample was normalized as a percentage relative to the kinase activity of SIK3 (wild-type). Each assay was performed in duplicate and repeated three times for biological replicates.

### Immunoprecipitation assay

Twenty-four hours after transfection, cells were processed with lysis buffer followed by the same rotating procedure with in vitro kinase assay. The supernatant was either used for immunoprecipitation or processed with a 6× sample buffer. The immunoprecipitation for 3xFLAG-tagged SIK3 proteins was performed using DDDDK-tagged Protein PURIFICATION KIT (MBL) according to the manufacturer’s procedure. The final immunoprecipitants were mixed with 6× sample buffer followed by the subjection to western blotting.

### SDS-PAGE, Phos-tag SDS-PAGE, and immunoblotting

The cell lysis for in vitro kinase assay, Phos-tag SDS-PAGE, and immunoprecipitation assay were processed similarly as described in vitro kinase assay section. The lysates were rotated for at least 15 minutes at 4°C, and then centrifuged at 14 000 *g* at 4°C for 12 minutes. The supernatant was mixed with 6× sample buffer and boiled for 5 minutes at 95°C. The samples were separated by SDS-PAGE and subsequently transferred to the PVDF membrane according to standard protocols. For Phos-tag SDS-PAGE, the samples were mixed with 1 mM MnCl_2_ followed by the separation by SDS-PAGE using polyacrylamide gels containing 50 μM Phos-tag acrylamide and 100 μM MnCl_2_. The proteins were transferred to the PVDF membrane with a transfer buffer with 0.1% SDS. Blots were incubated overnight at 4°C with a primary antibody in Can Get Signal Solution 1 (TOYOBO) or TBST with 5% bovine serum albumin or skim milk. The following antibodies were used as the primary antibody; anti-DYKDDDD tag antibody (mouse, monoclonal, Wako #1E6) as anti-FLAG antibody, anti-β-tubulin antibody (rabbit, monoclonal, Cell Signaling Technology #2128), anti-SIK3 phospho-T221 antibody (rabbit, polyclonal, Eurofins custom-made service using GQLLKpTWCGSPP peptide as an immunogen), anti-SIK3 phospho-S551 antibody (rabbit, polyclonal, Eurofins custom-made service using LGRRApSDGGAN peptide as an immunogen), anti-14-3-3 (pan) antibody (rabbit, polyclonal, Cell Signaling Technology #8312), anti-LKB1 (D60C5) antibody (rabbit, monoclonal, Cell Signaling Technology #3047), anti-GAPDH (D16H11) XP antibody (rabbit, monoclonal, Cell Signaling Technology #5174). After incubation with the primary antibody, the blots were washed with TBST and incubated with the following secondary antibody diluted in Can Get Signal Solution 2 or TBST with 5% bovine serum albumin or skim milk; horseradish peroxidase (HRP)-conjugated, anti-mouse IgG antibody (rabbit, polyclonal, Jackson ImmunoResearch Laboratories AB_2340066), HRP-conjugated, anti-rabbit IgG antibody (donkey, polyclonal, Jackson ImmunoResearch Laboratories AB_10015282). After incubation with the secondary antibody, the blots were washed with TBST and exposed to Clarity Western ECL Substrate (Bio-Rad). Chemiluminescence signaling was detected using FUSION Solo 6S.EDGE (Vilber-Lourmat). Band intensity was measured using ImageJ. Ratios of band intensities were calculated after subtraction of the band intensity of the vector sample as the background signal. Rf values were calculated as the migration length of electrophoresis.

### Luciferase reporter assay

Forty-eight hours after transfection, cells were processed with the passive lysis buffer (Promega). Following the cell lysis, luciferase activity was assessed using a Dual-Luciferase Reporter Assay System (Promega). The luminescence was measured using Infinite M Plex (TECAN). Each assay was performed in triplicate and repeated three times for biological replicates. Firefly luciferase activity was normalized to *Renilla* luciferase activity for each well.

### EEG/electromyogram electrode implantation surgery

At 8–10 weeks of age, male mice were implanted with EEG/electromyogram (EMG) electrodes under anesthesia with isoflurane. Each mouse was implanted with an EEG/EMG electrode containing four gold-plated metal electrode pins (0.50 × 0.40 × 2.60 mm) and two flexible Teflon-coated stainless steel wires (15-mm-long) as reported previously [29, 30]. The electrode pins were lowered under stereotaxic control, and the electrode socket was subsequently attached to the skull with dental cement (3M ESPE, RelyX U200 Unicem Syringe Dental Resin Cement). Of the four inserted metal electrode pins, two ipsilateral pins (anterior-posterior (AP): 0.50 mm, medial-lateral (ML): −1.27 mm, dorsal-ventral (DV): −1.3 mm; and AP: −4.53 mm, ML: −1.27 mm, DV: −1.3 mm) were used for EEG recording. For EMG recording, two stainless steel wires were inserted into the neck extensor muscles. All mice were allowed at least 7 days for recovery from the surgery. After the recovery, all mice were attached to a tether cable with a counterbalanced arm (Instech Laboratories) and then allowed free movement to habituate the recording conditions at least for 9 days.

### EEG/EMG recording and sleep/wakefulness analysis

EEG/EMG signals were obtained and analyzed as previously described with some modifications [29]. The EEG/EMG signals were amplified, filtered (EEG: 0.5–100 Hz; EMG: 5–300 Hz) with a multichannel amplifier (NIHON KODEN, #AB-611J), and digitized at a sampling rate of 250 Hz using an analog-to-digital converter (National Instruments #PCI-6220) and LabView (National Instruments)-based custom-made software. The EEG/EMG data were visualized and semiautomatically analyzed by MATLAB (MathWorks)-based software followed by visual inspection. Each 4-second epoch was staged into wakefulness, NREMS, and REMS. Wakefulness was scored based on the presence of low amplitude, fast EEG activity, and high-amplitude, variable EMG activity. NREMS was characterized by high-amplitude, delta (1–4 Hz)-frequency EEG waves and low EMG tonus, whereas REMS was staged based on theta (6–9 Hz)-dominant EEG oscillations and EMG atonia. The total time spent in wakefulness, NREMS, and REMS was derived by summing the total number of 4-second epochs in each state. Mean episode durations were determined by dividing the total time spent in each state by the number of episodes of that state. Epochs that contained movement artifacts were included in the state totals. EEG signals were subjected to FFT analysis from 1 to 30 Hz with a 1-Hz bin using MATLAB-based custom software. Epochs with artifacts and epochs in which either one or both of the epochs preceding or following were in a different vigilance state were excluded from the power spectrum analysis [31]. The EEG power of each frequency bin was expressed as a percentage of the mean total EEG power over the sum of 15–30 Hz during all sleep/wake states. The delta range or the theta range was calculated as the average of the EEG power of 1–4 Hz or 6–9 Hz, respectively. NREMS delta density was calculated as the sum of EEG delta power during NREMS over the sum of 15–30 Hz during NREMS. Hourly NREMS delta density was expressed as the hourly average of the NREMS delta density for each 1-hour bin. Time course analysis of NREMS delta density was performed as described previously [15, 32]. The basal recordings during light phase and during dark phase were divided into 12 sections and 8 sections, respectively, ensuring that equal amounts of NREMS contributed to each interval. The mean NREMS delta density and the midpoint of each time interval were calculated.

For sleep deprivation, we performed sleep deprivation for 6 hours from the onset of the light phase, ZT0, by gentle handling [33]. During sleep deprivation, food and water were available. To calculate NREMS delta power change, the recording during light phase after sleep deprivation was divided into eight sections, and the mean NREMS delta power and the midpoint of each time interval were calculated. The mean NREMS delta power in each interval was normalized by the mean NREMS delta power during ZT9–12 of basal recording. Process S fitting to calculate time constants of increasing (*Ti*) and decreasing (*Td*) exponential saturation function was performed as previously described [15, 30] with some modifications. The upper asymptote was defined as the 95% level of the delta power during NREMS, while the lower asymptote was defined as the intercept of the delta power distribution between REMS and NREMS, which was calculated using kernel density estimation. The fitting was evaluated by the coefficient of determination. If the coefficient of determination was lower than 0, the data were excluded. As a result, we excluded the data of two wild-type mice, three *Sik3*^*T221E/+*^ mice, three *Sik3*^*T221E;Slp/+*^ mice, and one *Sik3*^*Slp/+*^ mice.

For analysis of brief awakenings [32, 34], we calculated the number of brief awakenings (wake episode lasting 16 seconds or less) per hour of NREMS during the first 3 hours and last 4 hours of light phase (ZT0–3, ZT8–12) under the basal condition and during the first 3 hours of recovery sleep after sleep deprivation (ZT6–9). The density of brief awakenings during ZT8–12 under the basal condition, when sleep need is the lowest, was subtracted from the densities of brief awakenings during ZT0–3 under the basal condition and during ZT6–9 after sleep deprivation, when sleep need is the highest.

### Analysis of peak onset of wakefulness

The onset of the peak of wakefulness was calculated as previously described [20] with some modifications. The sum of the wake time in 10-minute bin during basal recording in individual mice was calculated and then smoothed with a 4-hour moving average. Smoothed wake time was normalized by the highest value in each mouse. Then, the normalized values were binarized as above and below the mean of the normalized value. The onset of the peak of wakefulness was defined as the latency from ZT0 to the beginning of the longest consecutive period above the mean of the normalized values.

### Circadian behavioral analysis

Circadian behavioral analysis was performed as previously reported [5] with some modifications. Male mice, 8–15 weeks of age, were individually housed in a cage containing a wireless running wheel (Med Associates #ENV-047). Cages were placed in a chamber lit with green LED lights, which consume less energy and emit light in a spectrum that entrains mice to a light cycle [35]. The mice were habituated to the measurement environment under a 12-hour light:12-hour dark cycle for at least 7 days before recording. After the habituation, the number of wheel revolutions was recorded under the light–dark condition for 14 days followed by under the constant darkness for 21 days. Finally, the condition was returned to the original light–dark cycle, and the number of wheel revolutions was recorded for 7 days. The number of wheel revolutions of the wheels was obtained with a 1-minute bin using Wheel Manager software (Med Associates) via a receiving device (Med Associates #DIG807). The circadian period was calculated using a χ^2^ periodogram from days 5 to 21 under the constant darkness. The activity onset for wheel running was defined as the time when the difference in the total number of wheel revolutions between the 6 hours before and after a candidate time bin was the largest. The periodogram computation and actogram visualization were performed using Python.

### Statistical analysis

Statistical analyses were performed using Prism 10 (GraphPad). We used paired or unpaired *t* test for two-group comparisons, one-way ANOVA for multiple comparisons, and two-way ANOVA for multiple comparisons involving two independent variables. If there were no missing values, two-way repeated-measures ANOVA was used for time course or hourly analysis. If there were missing values, a mixed-effects model was used for hourly analysis. If one variable has two groups, we used Sidak’s test for a multiple comparison. If one variable has more than two groups, we used Tukey’s test for a multiple comparison. *P* < 0.05 was considered statistically significant. Data are presented as the mean ± SEM.

## Results

### SIK3 (T221E) has distinct characteristics in kinase activity and downstream transcription

First, we used several methods to characterize SIK3 (T221E) protein, a phosphomimetic mutant of T221, and to determine how the presence of SLP mutation on the T221E allele affects the properties of SIK3 (T221E) (Figure 1A). In vitro kinase assay showed that SIK3 (T221E) had reduced kinase activity to about 40%–50% of wild-type SIK3 and that SIK3 (T221A) lacked kinase activity as previously reported [8] (Figure 1B). Whereas SIK3 (SLP) and SIK3 (S551A) showed similar kinase activity with wild-type SIK3, SIK3 (T221E;SLP) had lower kinase activity than SIK3 (T221E). Since the readout of SIK3 signaling is HDAC4-dependent transrepression [36], we performed a luciferase assay measuring MEF2C-dependent transcriptional activity that is known to be repressed by nuclear HDAC4. The kinase-dead mutant SIK3 (T221A) did not show any effect on MEF2C-dependent transcription (Figure 1C). SIK3 (T221E) exhibited slightly but significantly higher MEF2C-dependent transcription than wild-type SIK3. SIK3 (SLP) showed higher MEF2C-dependent transcription than wild-type SIK3 as previously shown [8]. The presence of T221E substitution on SIK3 (SLP) resulted in increased MEF2C-dependent transcription. Thus, the strength of kinase activity using in vitro kinase assays does not necessarily explain differences in MEF2C-dependent transcription.

Next, we performed Phos-tag SDS-PAGE to examine the interdependence among multiple phosphorylation sites of SIK3. Wild-type SIK3 showed three major mobility bands with similar patterns (Figure 1D). Since Phos-tag slows down the mobility of phosphorylated forms, the highest mobility band or the lowest band corresponds to the nonphosphorylated form of SIK3. SIK3 (SLP) showed a decreased intensity of the upper band and an increased intensity of the middle band compared to wild-type SIK3, which may be due to the loss of the S551-containing PKA-phosphorylation site in SIK3 (SLP). SIK3 (T221A) mainly existed in a nonphosphorylated form (Figure 1D) and showed very low phosphorylation at S551 (Figure 1E), suggesting that the kinase activity of SIK3 is required for the phosphorylation at S551. SIK3 (T221A;SLP) lacked phosphorylated forms. SIK3 (T221E) showed similar mobilities as SIK3 (T221A) with increased intensity of the middle band (Figure 1D). Moreover, SIK3 (T221E) exhibited higher phosphorylation at S551 than wild-type SIK3 (Figure 1E). Immunoprecipitation using anti-FLAG antibody for FLAG-tag SIK3 variants exhibited that SIK3 (SLP) lacked 14-3-3 binding (Figure 1F) as previously reported [13]. SIK3 (T221A) showed a decreased 14-3-3 binding compared to wild-type SIK3 possibly due to the decreased S551 phosphorylation. Whereas wild-type SIK3 and SIK3 (T221E) did not show interaction with LKB1, SIK3 (T221A;SLP) and SIK3 (T221E;SLP) interacted with LKB1 (Figure 1F). Thus, SIK3 (T221E) exhibited lower in vitro kinase activity, slight but significant inhibition of HDAC4-dependent transrepression, and increased phosphorylation at S551, compared to wild-type SIK3.

### SIK3 (T221E) mice showed normal sleep time and responses to sleep deprivation

**Figure 2.**
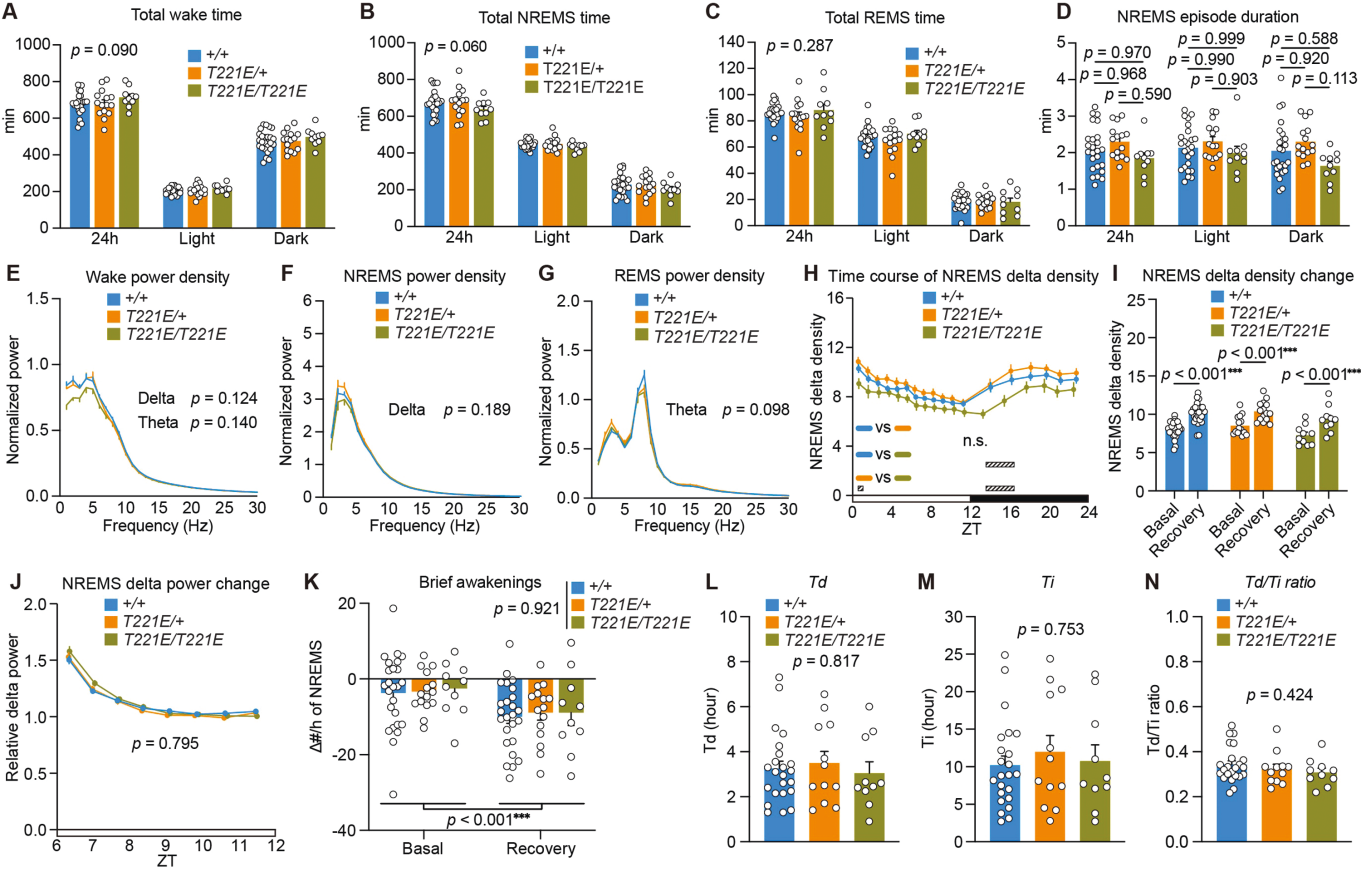
Sleep/wakefulness of SIK3 (T221E) mice. (A–D) Time spent in wakefulness (A), NREMS (B), REMS (C), and NREMS episode duration (D) over 24 hours and during light and dark phases of *Sik3*^*+/+*^ mice (*n* = 25), *Sik3*^*T221E/+*^ mice (*n* = 15), and *Sik3*^*T221E/T221E*^ mice (*n* = 10). Two-way ANOVA (A, B, C) followed by Tukey’s test (D). (E–H) EEG power spectra during wake (E), NREMS (F), REMS (G), and time course of NREMS delta density over 24 hours (H). One-way ANOVA (E–G) for delta and theta range in EEG power spectra. Two-way repeated-measures ANOVA followed by Tukey’s test (H). (I) NREMS delta density during ZT6–ZT8 under the basal condition and after 6-hour sleep deprivation. Paired *t* test. (J) NREMS delta power changes after sleep deprivation relative to that during ZT9–12 under the basal condition. Two-way repeated-measures ANOVA. (K) The changes in the densities of brief awakenings during ZT0–3 under the basal condition and during ZT6–9 after sleep deprivation compared to that during ZT8–12 under the basal condition. Two-way repeated-measures ANOVA, *p* values for genotype and time factors are shown. (L–N) *Td* (L), *Ti* (M), and the *Td*/*Ti* ratio (N) derived from Process S fitting for sleep need dynamics of *Sik3*^*+/+*^ mice (*n* = 23), *Sik3*^*T221E/+*^ mice (*n* = 12), and *Sik3*^*T221E/T221E*^ mice (*n* = 10). One-way ANOVA. Data are presented as the mean ± SEM. *p < 0.05, **p < 0.01, ***p < 0.001. n.s. indicates nonsignificance. Hatched rectangles in (H) indicate intervals with a significant difference between genotypes (*p *< 0.05).

Next, we examined the sleep/wake behavior of *Sik3*^*T221E*^ mice. Both heterozygous and homozygous *Sik3*^*T221E*^ mice showed similar time spent in wake, NREMS, REMS, and NREMS episode duration as the control group (Figure 2A–D). *Sik3*^*T221E*^ mice also showed similar EEG delta range power during wake and NREMS and theta range power during wake and REMS as the control group (Figure 2E–G). Homozygous *Sik3*^*T221E*^ mice showed a decreased NREMS delta density compared to wild-type and heterozygous *Sik3*^*T221E*^ mice, especially at the beginning of the dark phase (Figure 2H and Supplementary Figure S1A). After 6 hours of sleep deprivation with gentle handling from the beginning of the light phase, ZT0, *Sik3*^*T221E*^ mice exhibited an increase in NREMS delta power similar to wild-type mice (Figure 2I and J). Additionally, *Sik3*^*T221E*^ mice showed a similar density of brief awakenings, which is an indicator of consolidated sleep associated with sleep need [32, 34], under the basal condition and after sleep deprivation to wild-type mice (Figure 2K). To further investigate the dynamics of sleep need, we performed a curve fitting of the dynamics of Process S or sleep need to empirical delta power based on sleep/wake patterns of 48 hours including 6-hour sleep deprivation [15, 30]. The decreasing time constant (*Td*) and increasing time constant (*Ti*) of *Sik3*^*T221E*^ mice were similar to those of wild-type mice (Figure 2L–N). Thus, the substitution of the T221 for glutamic acid had only a minor impact on sleep/wakefulness.

### SIK3 (T221E) mice showed a delayed peak of wakefulness and a longer circadian period

**Figure 3.**
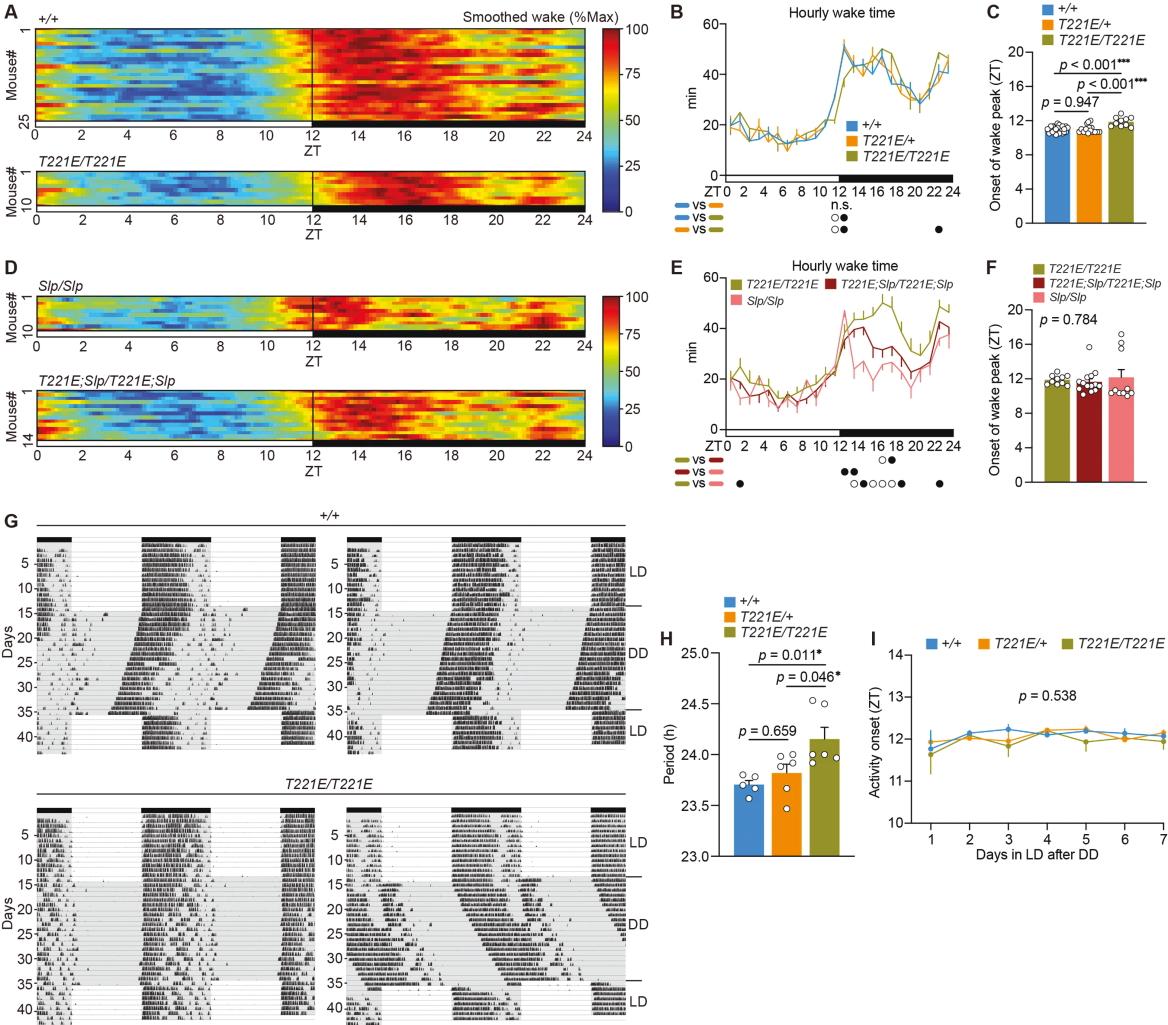
SIK3 (T221E) mice showed a delayed peak of wakefulness and a longer circadian period. (A) Heatmaps of smoothed wake time of individual *Sik3*^*+/+*^ mice and *Sik3*^*T221E/T221E*^ mice. (B) Circadian variation of wake time of *Sik3*^*+/+*^ mice, *Sik3*^*T221E/+*^ mice, and *Sik3*^*T221E/T221E*^ mice. (C) The onset of the peak of wakefulness in *Sik3*^*+/+*^ mice, *Sik3*^*T221E/+*^ mice, and *Sik3*^*T221E/T221E*^ mice. (D) Heatmaps of smoothed wake time of individual *Sik3*^*Slp/Slp*^ mice and *Sik3*^*T221E;Slp/T221E;Slp*^ mice. (E) Circadian variation of wake time of *Sik3*^*T221E/T221E*^ mice, *Sik3*^*T221E;Slp/T221E;Slp*^ mice, and *Sik3*^*Slp/Slp*^ mice. (F) The onset of the peak of wakefulness in *Sik3*^*T221E/T221E*^ mice, *Sik3*^*T221E;Slp/T221E;Slp*^ mice, and *Sik3*^*Slp/Slp*^ mice. (G) Representative actograms of *Sik3*^*+/+*^ mice (upper) and *Sik3*^*T221E/T221E*^ mice (below). (H) Circadian periods of *Sik3*^*T221E*^ mice under constant dark condition. (I) Activity onset of *Sik3*^*T221E*^ mice during re-entrainment to the original light–dark cycle after the constant darkness. Two-way repeated-measures ANOVA followed by Tukey’s test (B, E), one-way ANOVA followed by Tukey’s test (C, F, H), two-way repeated-measures ANOVA (I). Data are presented as the mean ± SEM. *p < 0.05, **p < 0.01, ***p < 0.001 or *p *< 0.05 (filled circle), *p *< 0.01 (open circle). n.s. indicates nonsignificance.

Next, we examined diurnal changes in sleep/wakefulness and circadian behavior of *Sik3*^*T221E*^ mice. Wild-type mice exhibited a rapid increase in wakefulness at the beginning of the dark phase while homozygous *Sik3*^*T221E*^ mice exhibited a delayed onset and slow increase in wakefulness after ZT11 (Figure 3A and B). Hourly wake time of homozygous *Sik3*^*T221E*^ mice was smaller than that of heterozygous *Sik3*^*T221E*^ mice and wild-type mice for 2 hours from ZT11 (Figure 3B). Homozygous *Sik3*^*T221E*^ mice exhibited a delayed peak of wakefulness at the beginning of the dark phase (Figure 3C).

When circadian rhythm was assessed using a running wheel-installed home cage under constant darkness, homozygous *Sik3*^*T221E*^ mice exhibited a longer circadian period than wild-type and heterozygous *Sik3*^*T221E*^ mice (Figure 3G and H). Similar to *Sik3*^*+/+*^ mice, the activity onset of homozygous *Sik3*^*T221E*^ mice was immediately adjusted to the beginning of the dark phase after the release to the original light–dark cycles (Figure 3G and I), which may be explained by the masking effect of light. Thus, homozygous *Sik3*^*T221E*^ mice showed a slow increase in wakefulness at the early dark phase, a longer circadian period, and an intact masking effect of light.

### *T221E* substitution alleviated sleep changes due to the *Slp* mutation

**Figure 4.**
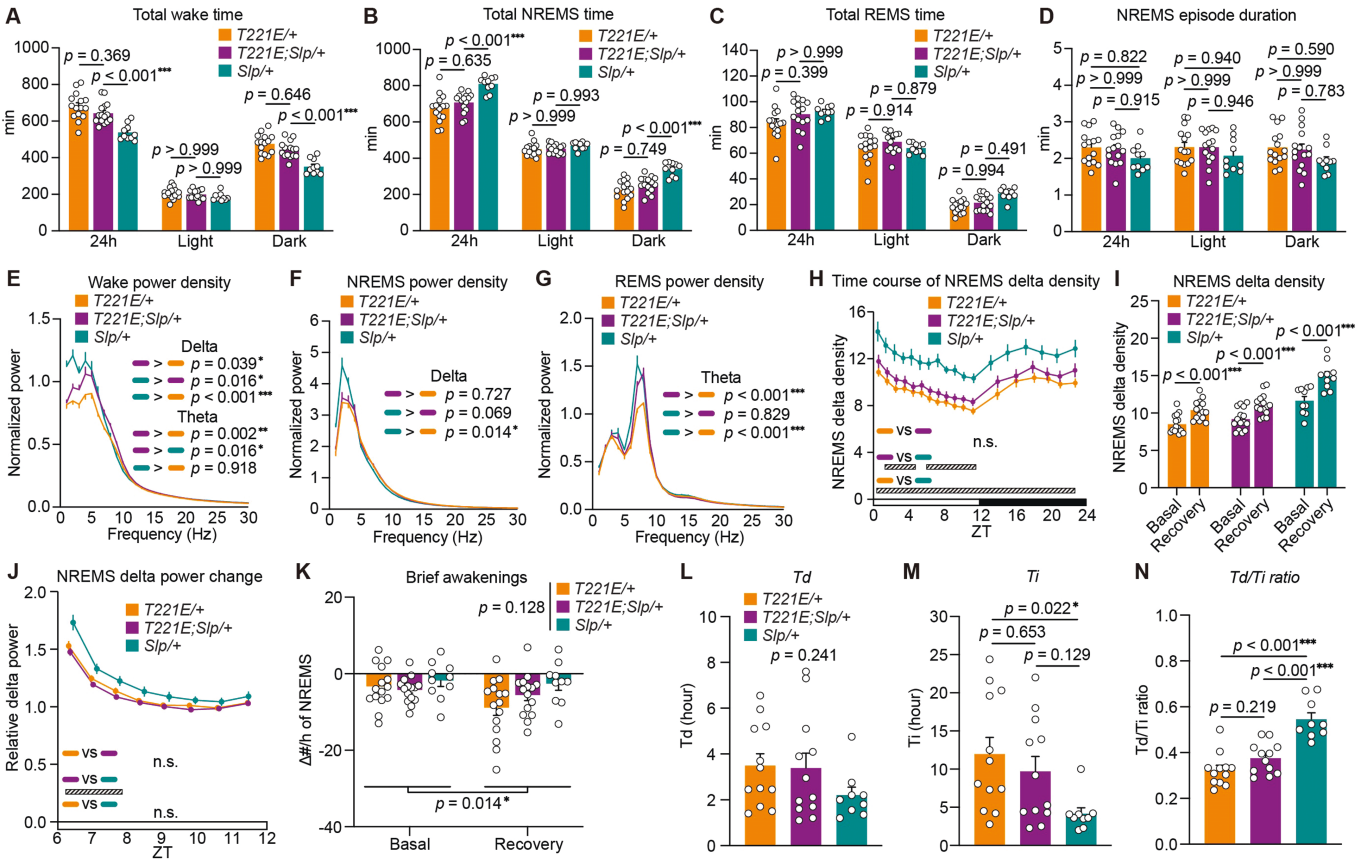
*T221E* substitution on the *Slp* allele alleviated sleep changes due to the *Slp* mutation. (A–D) Time spent in wake (A), NREMS (B), REMS (C), and episode duration in NREMS (D) over 24 hours and during light and dark phases of *Sik3*^*T221E/+*^ mice (*n* = 15), *Sik3*^*T221E;Slp/+*^ mice (*n* = 15), and *Sik3*^*Slp/+*^ mice (*n* = 10). Two-way ANOVA followed by Tukey’s test (A–D). (E–H) EEG power spectra during wake (E), NREMS (F), REMS (G), and time course of NREMS delta density over 24 hours (H). One-way ANOVA followed by Tukey’s test (E–G) for delta and theta range in EEG power spectra. Two-way repeated-measures ANOVA followed by Tukey’s test (H). (I) NREMS delta density during ZT6–ZT8 under the basal condition and after 6-hour sleep deprivation. Paired *t* test. (J) NREMS delta power changes after sleep deprivation relative to that during ZT9–12 under the basal condition. Two-way repeated-measures ANOVA followed by Tukey’s test. (K) The changes in the densities of brief awakenings during ZT0–3 under the basal condition and during ZT6–9 after sleep deprivation compared to that during ZT8–12 under the basal condition. Two-way repeated-measures ANOVA, *p* values for genotype and time factors are shown. (L–N) *Td* (L), *Ti* (M), and the *Td*/*Ti* ratio (N) derived from Process S fitting for sleep need dynamics of *Sik3*^*T221E/+*^ mice (*n* = 12), *Sik3*^*T221E;Slp/+*^ mice (*n* = 12), and *Sik3*^*Slp/+*^ mice (*n* = 9). One-way ANOVA (L) followed by Tukey’s test (M, N). Data are presented as the mean ± SEM. *p < 0.05, **p < 0.01, ***p < 0.001. n.s. indicates nonsignificance. Hatched rectangles in (H) and (J) indicate intervals with a significant difference between genotypes (*p *< 0.05).

Next, we examined how the presence of *T221E* substitution on the *Slp* allele affects the long-sleeper phenotype of *Sik3*^*Slp*^ mice. Heterozygous *Sik3*^*T221E;Slp*^ mice showed longer wake time and shorter NREMS time than heterozygous *Sik3*^*Slp*^ mice (Figure 4A and B), whereas REMS time and NREMS episode duration of heterozygous *Sik3*^*T221E;Slp*^ mice were similar to those of heterozygous *Sik3*^*Slp*^ mice (Figure 4C and D). Delta power during wake in heterozygous *Sik3*^*T221E;Slp*^ mice was smaller than in *Sik3*^*Slp*^ mice (Figure 4E), which may indicate the decreased synchronization of local cortical neurons reflecting decreased sleep need [13, 37]. Time course of NREMS delta power of heterozygous *Sik3*^*T221E;Slp*^ mice was smaller than that of heterozygous *Sik3*^*Slp*^ mice and similar to that of heterozygous *Sik3*^*T221E*^ mice (Figure 4F and H and Supplementary Figure S1B). Theta range power during wake was higher in heterozygous *Sik3*^*T221E;Slp*^ mice than in heterozygous *Sik3*^*T221E*^ and *Sik3*^*Slp*^ mice (Figure 4E). Theta range power during REMS in heterozygous *Sik3*^*T221E*^ mice was smaller than that in heterozygous *Sik3*^*T221E;Slp*^ and *Sik3*^*Slp*^ mice (Figure 4G). After sleep deprivation for 6 hours, heterozygous *Sik3*^*T221E;Slp*^ mice showed increased NREMS delta range power, which was smaller than heterozygous *Sik3*^*Slp*^ mice (Figure 4I and J). The density of brief awakenings under the basal condition and after sleep deprivation were not different among heterozygous *Sik3*^*T221E*^, *Sik3*^*T221E;Slp*^, and *Sik3*^*Slp*^ mice (Figure 4K). Heterozygous *Sik3*^*T221E;Slp*^ mice showed a lower *Td*/*Ti* ratio than heterozygous *Sik3*^*Slp*^ mice and similar to *Sik3*^*T221E*^ mice (Figure 4L–N). Thus, the presence of *T221E* substitution on the *Slp* allele alleviated the long-sleeper phenotype of heterozygous *Sik3*^*Slp*^ mice.

**Figure 5.**
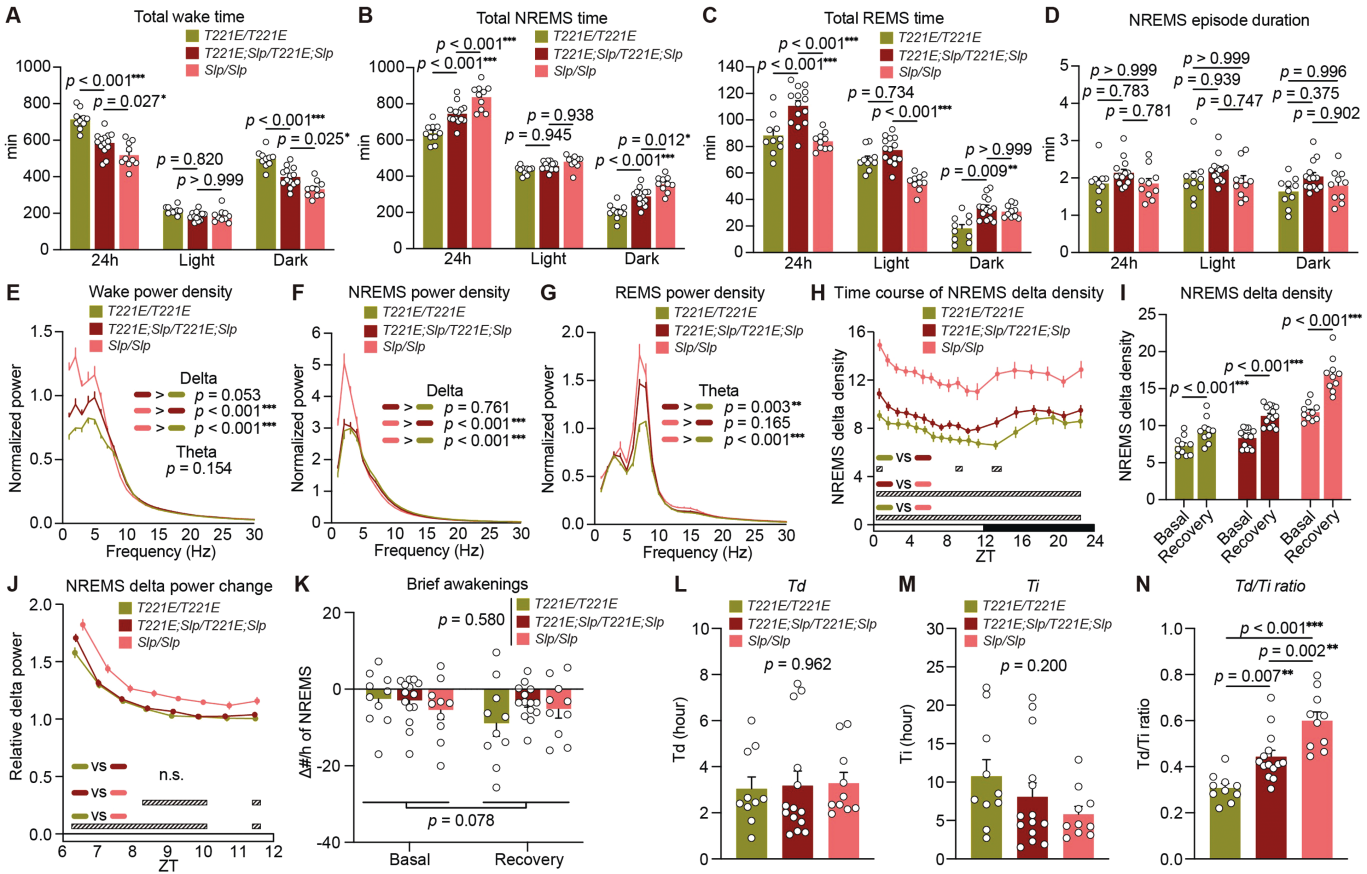
Sleep/wakefulness of homozygous SIK3 (T221E;SLP) mice. (A–D) Time spent in wake (A), NREMS (B), REMS (C), and episode duration in NREMS (D) over 24 hours and during light and dark phases of *Sik3*^*T221E/T221E*^ mice (*n* = 10), *Sik3*^*T221E;Slp/T221E;Slp*^ mice (*n* = 14), and *Sik3*^*Slp/Slp*^ mice (*n* = 10). Two-way ANOVA followed by Tukey’s test (A–D). (E–H) EEG power spectra during wake (E), NREMS (F), REMS (G), and time course of NREMS delta density over 24 hours (H). One-way ANOVA followed by Tukey’s test (E–G) for delta and theta range in EEG power spectra. Two-way repeated-measures ANOVA followed by Tukey’s test (H). (I) NREMS delta density during ZT6–ZT8 under the basal condition and after 6-hour sleep deprivation. Paired *t* test. (J) NREMS delta power changes after sleep deprivation relative to that during ZT9–12 under the basal condition. Two-way repeated-measures ANOVA followed by Tukey’s test. (K) The changes in the densities of brief awakenings during ZT0–3 under the basal condition and during ZT6–9 after sleep deprivation compared to that during ZT8–12 under the basal condition. Two-way repeated-measures ANOVA, *p* values for genotype and time factors are shown. (L–N) *Td* (L), *Ti* (M), and the *Td*/*Ti* ratio (N) derived from Process S fitting for sleep need dynamics of *Sik3*^*T221E/T221E*^ mice (*n* = 10), *Sik3*^*T221E;Slp/T221E;Slp*^ mice (*n* = 14), and *Sik3*^*Slp/Slp*^ mice (*n* = 10). One-way ANOVA (L, M) followed by Tukey’s test (N). Data are presented as the mean ± SEM. *p < 0.05, **p < 0.01, ***p < 0.001. n.s. indicates nonsignificance. Hatched rectangles in (H) and (J) indicate intervals with a significant difference between genotypes (*p *< 0.05).

Homozygous *Sik3*^*T221E;Slp*^ mice showed longer wake time and shorter NREMS time than homozygous *Sik3*^*Slp*^ mice with similar NREMS episode duration (Figure 5A, B, and D). Homozygous *Sik3*^*T221E;Slp*^ mice showed longer REMS time than homozygous *Sik3*^*Slp*^ mice (Figure 5C). Homozygous *Sik3*^*T221E;Slp*^ mice showed a lower delta power during wake and NREMS than homozygous *Sik3*^*Slp*^ mice (Figure 5E, F, and H and Supplementary Figure S1C). While theta range power during wake was similar (Figure 5E), theta range power during REMS was higher in homozygous *Sik3*^*T221E;Slp*^ and *Sik3*^*Slp*^ mice than in homozygous *Sik3*^*T221E*^ mice (Figure 5G). Homozygous *Sik3*^*T221E;Slp*^ mice showed a smaller increase in NREMS delta power in response to sleep deprivation (Figure 5I and J) with a similar microarousal density (Figure 5K) and a smaller *Td*/*Ti* ratio than homozygous *Sik3*^*Slp*^ mice (Figure 5L–N). In terms of circadian-related behavior, homozygous *Sik3*^*T221E;Slp*^ mice did not show a significant difference in the onset of the peak of wakefulness compared to homozygous *Sik3*^*Slp*^ mice despite a tendency for delay (Figure 3D–F). This may be because the presence of the *Slp* mutation shortened wake time, making the *T221E*-induced attenuation of wake onset less detectable. Thus, the *T221E* substitution reduced NREMS time prolongation, NREMS delta power increase, and *Td*/*Ti* ratio increase in mice with the heterozygous and homozygous *Slp* mutation in the *Sik3* gene.

Although sleep/wake behavior of heterozygous *Sik3*^*T221E;Slp*^ mice was similar to that of heterozygous *Sik3*^*T221E*^ mice (Figure 4), homozygous *Sik3*^*T221E;Slp*^ mice showed a shorter wake time and a longer NREMS time and REMS time than homozygous *Sik3*^*T221E*^ mice (Figure 5A–C). Homozygous *Sik3*^*T221E;Slp*^ mice showed a slight increase in NREMS delta density under the basal condition compared to homozygous *Sik3*^*T221E*^ mice, while their response to sleep deprivation was comparable (Figure 5F, H, and J and Supplementary Figure S1C). *Td/Ti* ratio of homozygous *Sik3*^*T221E;Slp*^ mice was higher than that of homozygous *Sik3*^*T221E*^ mice (Figure 5L and M). Thus, homozygous *Slp* mutation in *T221E* allele in the *Sik3* gene induced an increase of sleep time and an enhancement of sleep need.

**Figure 6.**
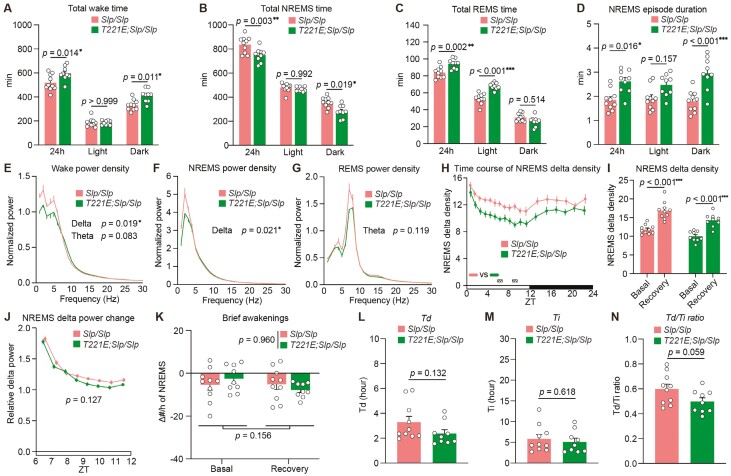
Heterozygous *T221E* substitution alleviated sleep changes due to the homozygous *Slp* mutation. (A–D) Time spent in wake (A), NREMS (B), REMS (C), and episode duration in NREMS (D) over 24 hours and during light and dark phases of *Sik3*^*Slp/Slp*^ mice (*n* = 10) and *Sik3*^*T221E;Slp/Slp*^ mice (*n* = 9). Two-way ANOVA followed by Tukey’s test (A–D). (E–H) EEG power spectra during wake (E), NREMS (F), REMS (G), and time course of NREMS delta density over 24 hours (H). Unpaired *t* test (E–G) for delta and theta range in EEG power spectra. Two-way repeated-measures ANOVA followed by Sidak’s test (H). (I) NREMS delta density during ZT6–ZT8 under the basal condition and after 6-hour sleep deprivation. Paired *t* test. (J) NREMS delta power changes after sleep deprivation relative to that during ZT9–12 under the basal condition. Two-way repeated-measures ANOVA. (K) The changes in the densities of brief awakenings during ZT0–3 under the basal condition and during ZT6–9 after sleep deprivation compared to that during ZT8–12 under the basal condition. Two-way repeated-measures ANOVA, *P* values for genotype and time factors are shown. (L–N) *Td* (L), *Ti* (M), and the *Td*/*Ti* ratio (N) derived from Process S fitting for sleep need dynamics of *Sik3*^*Slp/Slp*^ mice (*n* = 10) and *Sik3*^*T221E;Slp/Slp*^ mice (*n* = 9). Unpaired *t* test (L–N). Data are presented as the mean ± SEM. *p < 0.05, **p < 0.01, ***p < 0.001. Hatched rectangles in (H) indicate intervals with a significant difference between genotypes (*p *< 0.05).

We further examined the effect of one allele of *T221E* substitution under the homozygous *Slp* mutation. *Sik3*^*T221E;Slp/Slp*^ mice exhibited increased wake time, decreased NREMS time, and increased REMS time with longer NREMS episode duration compared to *Sik3*^*Slp/Slp*^ mice (Figure 6A–D). *Sik3*^*T221E;Slp/Slp*^ mice exhibited decreased delta power during wake and NREMS and similar theta power during wake and REMS compared to *Sik3*^*Slp/Slp*^ mice (Figure 6E–H and Supplementary Figure S1D). After sleep deprivation, NREMS delta power change and microarousal density of *Sik3*^*T221E;Slp/Slp*^ mice were similar to that of *Sik3*^*Slp/Slp*^ mice (Figure 6I–K). *Sik3*^*T221E;Slp/Slp*^ mice showed a similar *Td*/*Ti* ratio compared to *Sik3*^*Slp/Slp*^ mice (Figure 6L–N). Thus, one allele of *T221E* substitution is sufficient to alleviate increased NREMS time and quality due to the homozygous *Slp* mutation.

## Discussion

The present study demonstrated that the phosphomimetic substitution of T221 with glutamic acid did not affect the amount and quality of NREMS but affected the timing of wakefulness and circadian period length. We also showed that *T221E* substitution alleviated sleep changes due to the *Slp* mutation.

**Figure 7.**
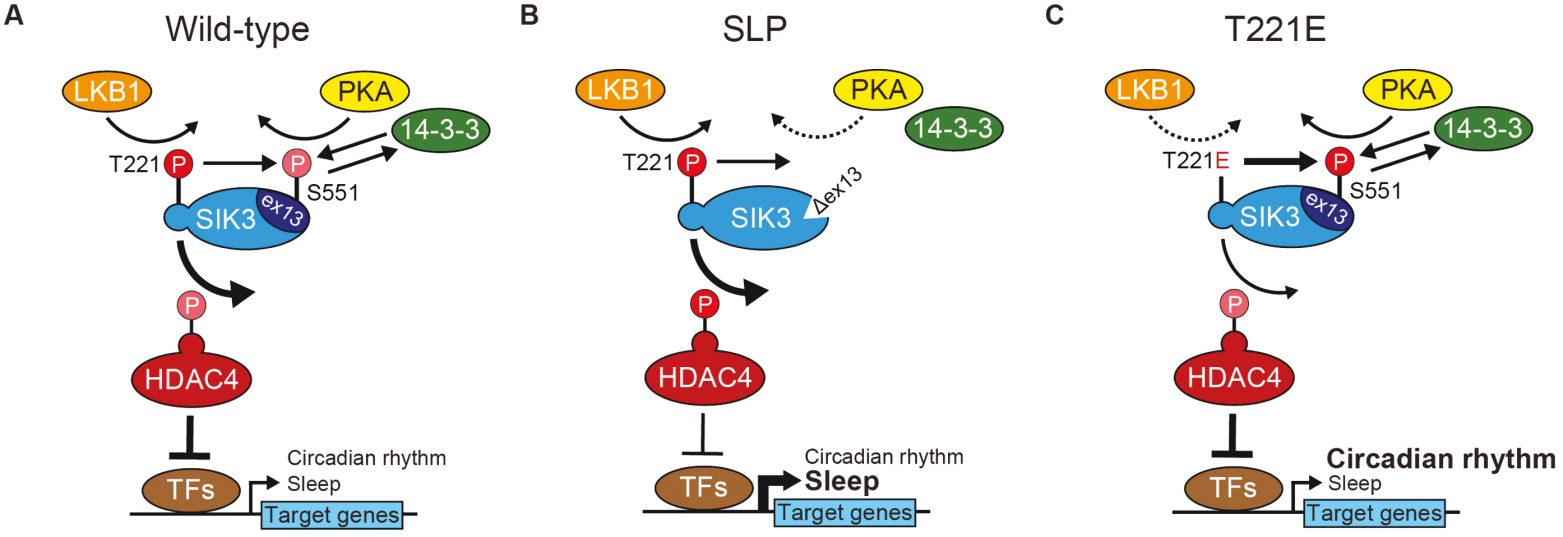
Scheme summarizing the relationship between two phosphorylation sites in the regulation of SIK3 activity and sleep/wakefulness. (A) LKB1 and PKA phosphorylate T221 and S551, respectively. 14-3-3 binds to phosphorylated S551. When SIK3 phosphorylates HDAC4, phosphorylated HDAC4 cannot bind to transcription factors (TFs) and transcription is no longer repressed. Phosphorylation of T221 is required for phosphorylation of S551. In wild-type mice, SIK3 promotes NREMS by repressing HDAC4-mediated transcriptional repression of sleep-promoting genes. (B) SIK3 (SLP) protein lacks the ability to bind to 14-3-3 and exerts enhanced suppression on HDAC4-mediated transcriptional repression. In *Sik3*^*Slp*^ mice, HDAC4-mediated transcriptional repression of sleep-promoting genes is more suppressed, resulting in an increase in NREMS delta power and time. (C) SIK3 (T221E) protein has constitutive but weaker kinase activity than wild-type SIK3. In *Sik3*^*T221E*^ mice, lower kinase activity and reduced regulation by LKB1 primarily affect circadian behavior and sleep/wake patterns, rather than NREMS amount or NREMS delta power.

We have previously shown that the LKB1-SIK3-HDAC4 pathway regulates the amount and quality of NREMS in mice [7, 8] (Figure 7A). SIK3 (SLP) protein exhibited increased MEF2C-dependent transcription, lacked the ability to bind 14-3-3, and had kinase activity comparable to wild-type SIK3 protein assessed by in vitro kinase assay (Figure 7B). Mice expressing SIK3 (SLP) had longer NREMS time and higher NREMS delta power than wild-type mice, and exhibited similar wake patterns and circadian behavior as wild-type mice [5]. In the present study, SIK3 (T221E) protein showed lower kinase activity assessed by in vitro kinase assay than wild-type SIK3 protein, and a slightly higher but generally similar level of MEF2C-dependent transcription (Figure 7C). *Sik3*^*T221E*^ mice showed similar NREMS time and NREMS delta power as wild-type mice and exhibited delayed onset of wakefulness and longer circadian period length than wild-type mice.

The effect of single or dual phosphorylation at T221 and S551 depends on the type of assay. In vivo or cell-based assay, such as the luciferase transcriptional assay, generally shows a diminished influence of scaffold proteins like 14-3-3 compared to in vitro kinase assay. This difference may explain why the gain-of-function characteristics, or enhanced NREMS time and delta density, observed in *Sik3*^*Slp*^ mice or *Sik3*^*S551A*^ mice are more accurately replicated by the luciferase transcriptional assay than by in vitro kinase assay. Conversely, the less pronounced changes in NREMS time and delta density in *Sik3*^*T221E;Slp*^ mice compared to *Sik3*^*Slp*^ mice are better reflected in in vitro kinase assay than in luciferase transcriptional assay. In other words, the effect of phosphorylation at S551 on sleep is more consistent with assays involving 14-3-3 interaction, whereas the effect of T221 phosphorylation on sleep correlates closely with assays directly assessing the kinase activity of SIK3 protein. Since the kinase activity of SIK3 is required for the sleep-promoting effect of the SLP mutation [8, 19], a reduction in the kinase activity due to the presence of T221E substitution may explain the alleviation of the sleep phenotype by the SLP mutation in *Sik3*^*T221E;Slp*^ mice.

Although SIK3 (T221E;SLP) protein is independent of LKB1 regulation and lacks a PKA-phosphorylation site S551, homozygous *Sik3*^*T221E;Slp*^ mice showed a rebound of NREMS delta power after sleep deprivation. The conserved homeostatic sleep regulation in homozygous *Sik3*^*T221E;Slp*^ mice may be attributed to the presence of another PKA site 469th threonine residue, an additional regulatory site impacting SIK3 activity [17, 18], or compensatory mechanisms by other molecules.

Unexpectedly, we found that phosphorylation at T221, or kinase activity, is required for S551 phosphorylation, and that increased S551 phosphorylation in SIK3 (T221E). Given that S551 phosphorylation acts to inhibit the downstream transcription of SIK3, SIK3 kinase may prevent SIK3 itself from overacting downstream by exerting negative feedback via S551 phosphorylation. However, the underlying mechanism of this phosphorylation pathway and its physiological functions related to sleep warrant further investigations.

As we previously showed, SIK3 signaling pathway regulates the amount and quality of NREMS and the circadian behavior through forebrain excitatory neurons and SCN inhibitory neurons, respectively [8, 20]. Therefore, the changes in NREMS and circadian behavior observed in *Sik3*^*T221E*^ mice and *Sik3*^*T221E;Slp*^ mice may be due to altered SIK3 signaling in glutamatergic neurons in the forebrain and GABAergic neurons in the SCN, respectively. The differential effects of the same SIK3 modification on sleep or circadian behavior are thought to be explained by differences in the balance of LKB1 and PKA regulation and target genes of SIK3 downstream [8, 38], depending on neurons in which SIK3 works (Figure 7).

In contrast to the negligible alterations observed in the quantity and quality of NREMS, homozygous *Sik3*^*T221E*^ mice exhibited a delayed increase in wakefulness around the beginning of the dark phase and a longer circadian length compared to wild-type mice. These changes are consistent with those observed in mice lacking SIK3 in the SCN [20]. Given that the kinase activity of SIK3 (T221E) is significantly reduced compared to wild-type, circadian regulation by SIK3 in the SCN is thought to be sensitive to a reduction in its kinase activity. Since the kinase activity of SIK3 (T221E) is independent of phosphorylation by LKB1 [21], the main cause of the circadian-related changes in homozygous *Sik3*^*T221E*^ mice may not be reduced kinase activity but rather a lack of regulation by LKB1. The expression of LKB1 can be regulated by DEC1 [39], a transcription factor expressed in the SCN and involved in circadian rhythm regulation [40].

Theta power during REMS in heterozygous and homozygous *Sik3*^*T221E*^ mice was lower than that in heterozygous and homozygous *Sik3*^*Slp*^ mice and similar to that of wild-type mice. Theta power during REMS in *Sik3*^*T221E;Slp*^ mice was comparable to that observed in *Sik3*^*Slp*^ mice, rather than in *Sik3*^*T221E*^ mice. Similarly, overexpression of SIK3 (T221E;SLP) in neurons altered theta power during REMS [19]. These findings indicate that disturbed kinase activity by the T221E substitution has little effect on the increase in theta power during REMS due to the SLP mutation. In contrast, delta power during NREMS in *Sik3*^*T221E;Slp*^ mice was comparable to that observed in *Sik3*^*T221E*^ mice, rather than in *Sik3*^*Slp*^ mice, which suggests that disturbed kinase activity is critical to increase delta power due to the SLP mutation. Thus, SIK3 may regulate delta power during NREMS and theta power during REMS through distinct molecular mechanisms.

In summary, alterations in the kinase activity of SIK3 differentially affect sleep and circadian behavior. Rapid wakefulness at the onset of the dark phase is sensitive to the phosphomimetic mutation of T221, suggesting that SIK3 can be a therapeutic target for morning wake disturbances.

## Supplementary Material

Supplementary material is available at *SLEEP* online.

zsae279_suppl_Supplementary_Figure

## Data Availability

The data underlying this article will be shared on reasonable request to the corresponding author.
