# Supplementary material for: Partial activation of salt-inducible kinase 3 delays the onset of wakefulness and alleviates hypersomnia due to the lack of protein kinase A-phosphorylation site
Source: Sleep. 2024 Dec 4;48(2):zsae279. doi: 10.1093/sleep/zsae279 (PMC11807893; doi:10.1093/sleep/zsae279)
Supplement: zsae279_suppl_Supplementary_Figure [file zsae279_suppl_supplementary_figure.pdf]

Supplementary Figure

Partial activation of SIK3 delays the onset of wakefulness and alleviates hypersomnia due to the lack of protein kinase A-phosphorylation site

Shinya Nakata[1], Tomoyuki Fujiyama[1], Fuyuki Asano[1], Haruna Komiya[1], Noriko Hotta-Hirashima[1], Motoki Juichi[1], Daiki Komine[1], Miyo Kakizaki[1], Aya Ikkyu[1], Seiya Mizuno[2], Satoru Takahashi[2], Chika Miyoshi[1], Hiromasa Funato[1,3], Masashi Yanagisawa[1,4,5]

[1]International Institute for Integrative Sleep Medicine (WPI-IIIS), University of Tsukuba, Tsukuba, Ibaraki, Japan. [2]Laboratory Animal Resource Center and Transborder Medical Research Center, University of Tsukuba, Tsukuba, Ibaraki, Japan. [3]Department of Anatomy, Graduate School of Medicine, Toho University, Tokyo, Japan. [4]Department of Molecular Genetics, University of Texas Southwestern Medical Center, Dallas, TX, USA. [5]Life Science Center for Survival Dynamics, Tsukuba Advanced Research Alliance, University of Tsukuba, Tsukuba, Ibaraki, Japan.

Corresponding authors:

Masashi Yanagisawa, MD, PhD. E-mail: yanagisawa.masa.fu@u.tsukuba.ac.jp

Hiromasa Funato. MD, PhD. E-mail funato.hiromasa.km@u.tsukuba.ac.jp

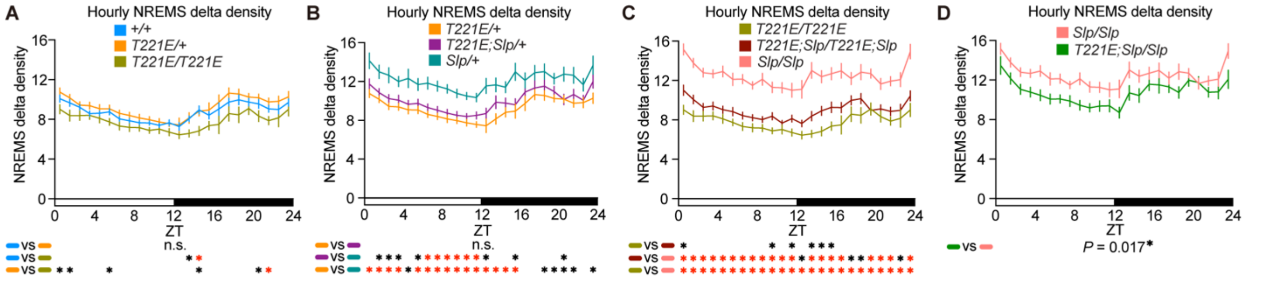

Supplementary Figure S1. Hourly NREMS delta density.

(A-D) Hourly NREMS delta density over a 24-hour period for each genotype comparison. Mixed-effects model (D) followed by Tukey's test (A, B, C). Data are presented as the mean ± SEM. *$P < 0.05$ (black), *$P < 0.01$ (red).
